# Possibilities and challenges for developing a successful vaccine for leishmaniasis

**DOI:** 10.1186/s13071-016-1553-y

**Published:** 2016-05-12

**Authors:** Saumya Srivastava, Prem Shankar, Jyotsna Mishra, Sarman Singh

**Affiliations:** Division of Clinical Microbiology and Molecular Medicine, Department of Laboratory Medicine, All India Institute of Medical Sciences, Ansari Nagar, New Delhi 110029 India

**Keywords:** Leishmaniasis, Immune response, Vaccine

## Abstract

Leishmaniasis is a vector-borne disease caused by different species of protozoan parasites of the genus *Leishmania*. It is a major health problem yet neglected tropical diseases, with approximately 350 million people worldwide at risk and more than 1.5 million infections occurring each year. Leishmaniasis has different clinical manifestations, including visceral (VL or kala-azar), cutaneous (CL), mucocutaneous (MCL), diffuse cutaneous (DCL) and post kala-azar dermal leishmaniasis (PKDL). Currently, the only mean to treat and control leishmaniasis is by rational medications and vector control. However, the number of available drugs is limited and even these are either exorbitantly priced, have toxic side effects or prove ineffective due to the emergence of resistant strains. On the other hand, the vector control methods are not so efficient. Therefore, there is an urgent need for developing a safe, effective, and affordable vaccine for the prevention of leishmaniasis. Although in recent years a large body of researchers has concentrated their efforts on this issue, yet only three vaccine candidates have gone for clinical trial, until date. These are: (i) killed vaccine in Brazil for human immunotherapy; (ii) live attenuated vaccine for humans in Uzbekistan; and (iii) second-generation vaccine for dog prophylaxis in Brazil. Nevertheless, there are at least half a dozen vaccine candidates in the pipeline. One can expect that, in the near future, the understanding of the whole genome of *Leishmania* spp. will expand the vaccine discovery and strategies that may provide novel vaccines. The present review focuses on the development and the status of various vaccines and potential vaccine candidates against leishmaniasis.

## Background

Leishmaniasis is a vector-borne disease caused by protozoan parasites belonging to the genus *Leishmania*. Leishmaniasis remained one of the world’s most neglected diseases until few years ago. It is prevalent in at least 88 countries across the tropical and subtropical regions of Africa, Asia, the Mediterranean, Southern Europe and South and Central Americas. Most of these (72 of 88) are developing countries. Approximately 12 million people are infected with a species of *Leishmania* at any given time point [1].

The causative agent, the *Leishmania* parasite, is transmitted through the bites of a tiny (2–3 mm long) female insect vectors, the phlebotomine sand flies. There are about 500 known *Phlebotomine* spp. but only 30 of these are incriminated in the transmission of leishmaniasis [2]. The female sand fly needs a blood meal for ovulation and egg development. While sucking the blood from an infected person or animal, the sand fly becomes infected with *Leishmania* spp. Over a period of 4 to 25 days, the parasites transform from the amastigote to promastigote stage and multiply in the gut of an infected sand fly vector. When the infected sand fly feeds on another person or animal, the promastigotes (an actively motile form of parasite) are inoculated in the new host and thus transmission cycle completes [2].

The clinical manifestations of leishmaniasis in humans depend on complex interactions between the virulence characteristics of the infecting *Leishmania* spp. and the immune responses of its host. The disease can manifest in a number of forms ranging from simple cutaneous ulcers to involvement of the liver and other visceral organs causing highly fatal visceral disease or kala-azar (KA). Accordingly, there are at least, three major clinical forms of the disease, the visceral leishmaniasis (VL), cutaneous leishmaniasis (CL) and mucocutaneous leishmaniasis (MCL). There are other forms also which include diffuse cutaneous leishmaniasis (DCL) and post kala-azar dermal leishmaniasis (PKDL) and these are often linked to host immune status as reviewed elsewhere [3]. For treatment of leishmaniasis the most commonly used drugs have been pentavalent antimonials, oral miltefosine, amphotericin B, liposomal amphotericin B and paromomycin. Major problems associated with these drugs are high cost, toxicity, duration of treatment, route of administration and the development of drug-resistant parasites [4].

However, taking clues from the fact that individuals once infected with *Leishmania* spp. after recovery become resistant to re-infections, efforts have been made to develop prophylactic vaccines [3, 5]. Although there is no licensed vaccine, as yet, against any form of leishmaniasis, a vaccine in principle should be possible. This assumption is based on the abundance of genetic and biological information available about the parasite [6]. Leishmaniasis is unique among parasitic diseases because a single vaccine could successfully prevent that disease and has the potential to protect against the infection caused by more than one species [6]. The studies of anti-leishmanial vaccine candidates have increased in recent years mainly after clarity of cell-mediated immune mechanisms for controlling the infection. However, current knowledge is mostly based on experimental animal models and cannot be extrapolated to humans or dogs [7]. The immune reactions against leishmaniasis are highly complex and while these may accelerate cure, some responses aggravate the disease. Both these responses depend on the particular stage of the disease, species of the infectious agent and host immune status [8]. Therefore, it becomes imperative to understand these pathophysiological and immunological complexities before trying to develop vaccines.

## Immunological paradigm in the host and the parasite

One of the most important aspects of *Leishmania* spp. infection is the ability of these parasites to evade and sabotage host immune responses. These characteristics also allow the parasite to persist and establish chronic infection. The protective immune response to *Leishmania* infection is predominantly cell-mediated immunity. The Th1 type immune response correlates with resistance, whereas Th2 response is associated with susceptibility to infection [9]. However, several factors influence resistance or susceptibility to leishmaniasis including genetic variation of the host, genetic variation of the parasites between species and strains, and chance factors such as the location, inoculum size and number of infective bites received by the host [10].

However, two major antigen presenting cells (APCs) which could be macrophages and/or dendritic cells (DCs) play critical roles in mediating the resistance and susceptibility during *Leishmania* infection. The macrophages and DCs play critical roles in the initiation, development, and maintenance of a protective immunity against *Leishmania* infection. However, the intracellular amastigotes, once internalized, can modulate cell-signalling pathways in macrophages by a variety of mechanisms resulting in the inhibition of cytokine responses and this can subvert the protective potential of these cells. These mechanisms include activation of inhibition of p38 mitogen-activated protein kinases [11], local activation of latent TGFb32 and NF-kB transactivation [12] and suppression of cytokine signalling (SOCS)-3 [13]. The production of IL-12 by DCs initiates Th1 response and protective immunity by promoting early NK cell activities, including IFN- γ production and cytotoxicity [14]. However, the exact mechanisms driving differentiation of naïve CD4+T cells into Th1 or Th2 phenotypes are still not very clear. One of the major mechanisms used by *Leishmania* parasite, to overcome the host immune response, is to inhibit the production of Th1-polarizing cytokine, IL-12, as well as preventing DCs from successfully presenting parasite antigens to T cells [15]. It has been shown that the turnover and activity of other immune cells, such as granulocytes, including neutrophil and eosinophil, are increased during the human VL [16].

### Current understanding of Th1/Th2 paradigm

Although our conventional understanding about Th1 *vs* Th2 response in leishmaniasis is still valid, the Th2 polarization has never been able to explain the severity of human leishmanial diseases. Recently, a number of other T cell subsets, including regulatory T (Treg) and Th17 cells, have been found to play important role in the susceptibility and resistance of both experimental and human leishmanial diseases. The Th17 and Treg cells are widely accepted subsets with important functions in the induction and control of the inflammatory response [17]. The Th17 cells in skin and mucosa play a vital role in the protection from several extracellular pathogens but also incriminated in the mediation of severe immune pathologies. It may be because these cells are involved in the recruitment, migration and activation of neutrophils [18]. IL-22 is also produced by Th17 cells, and to a lesser extent by natural killer and Th1 cells [18], which are particularly involved in immunity in epithelial and mucosal levels [19]. IL-17 and IL-22 are inflammatory cytokines that play a protective role against intracellular parasites such as *Leishmania* [20]. However, the role of IL-17 and IL-22 during leishmanial infection remains poorly defined [21]. Nevertheless, studies suggested that Th17-based cytokines may be associated with protection against leishmaniasis. Recently, Ghosh et al. demonstrated that administration of recombinant IL-17 and IL-23 caused a significant suppression of organ parasite burden in mice with a marked generation of IFN-γ and nitric oxide [22]. Along with signature Th1 cytokines, IL-17 and IL-22 were also found to have complementary roles in protection against visceral leishmaniasis and it was postulated that defects in the Th17 induction could increase the risk of visceral leishmaniasis [23]. Another cytokine IL­27 is related to IL­12 and is produced by antigen presenting cells, mainly macrophages and DCs. It is thought to be important in the regulation of Th17 cells [24] and induction of naïve human CD4 cells to IL­10 production [25]. In mice model, IL­27 is reported to be an important cytokine in the development of early Th1 response, and mediating suppression of early IL­4 burst [26].

The Treg cells are an important constituent of the immune system that suppresses or regulates immune responses of other cells. These cells can be found in many forms with the most well understood being those that express CD4, CD25, and Foxp3 (CD4+CD25+ regulatory T cells) ligands. The presence of these cells may, however, precondition the skin for survival of *Leishmania* parasites and favour long-­term parasite survival [27]. In humans, CD4+CD25+Treg cells are found in cutaneous lesions [28] and elevated intra lesional FoxP3 and IL-10 cells have been linked to disease progression in both murine and human *Leishmania* infection [29]. However, Th17 cytokines and the transcription factor FoxP3 have been scarcely studied in canine leishmaniasis [30–32]. The preliminary data point to antigen­induced IL­10 producing Foxp3­ T cells being responsible for delayed healing of cutaneous leishmaniasis [33, 34].

### Role of toll-like receptors and nucleotide-binding oligomerization domain

The activation of macrophage is first mediated by Toll-like receptors (TLRs), which play an important role in the control of infection [35]. It plays an essential role in linking the innate and adaptive immunity and enhances phagocytosis and killing process of the parasites. Therefore, it is involved in the first-line defence against *Leishmania* parasites by triggering NF-κB activation and downstream production of pro-inflammatory cytokines. Experimental models of visceral leishmaniasis support a protective role for TLR2, 4 and 9 in host immune responses to *Leishmania* infection [36]. TLR2 has been associated with a protective role in a number of murine models of leishmaniasis. In a *L. major* murine model, the absence of TLR2 led to an increased number of cutaneous lesions [37] and TLR2 and 3 were involved in the phagocytosis of *L. donovani* parasites [38]. TLRs bind to myeloid differentiation factor 88 (MyD88), a protein that interacts with several other molecules in a signalling cascade that leads to cytokines production [39]. It was also shown that TLR4 contributes to both innate and acquired immune responses during *L. major* infection [40]. The absence of TLR4 in a knockout murine *L. major* model resulted in increased cutaneous lesions [41] and treatment with TLR4 and TLR9 agonists lessened disease progression [42]. Tolouei and colleagues recently reported that the mean relative gene expression and membrane expression of TLR2 and TLR4 in the macrophages of patients with the healing form of cutaneous lesions were significantly higher than patients with the non-healing form of lesions. These findings advocate that there is a diverse role of both these TLRs in the outcome of CL lesions after *L. major* infection [43]. However, in human VL, Kumar et al. reported significantly higher levels of mRNA encoding both TLR2 and TLR4 in pre-treatment splenic aspirate samples, but no change in TLR9 was observed between these groups during *L. donovani* infection [44]. The downregulation of TLR3 in the lymph node has also been associated with the establishment of leishmaniasis. TLR3 is needed for nitric oxide production and phagocytosis of the parasite [38], therefore a downregulation of this TLR would favour disease progression. It has also suggested that TLR2 and TLR3 play an important role in recognition of *L. donovani* promastigote by IFNγ-primed macrophages by using RNA interference [38]. There are limited data available on the expression of these TLRs in human VL, particularly at sites of infection, such as the spleen. Recently, ex-vivo expression of TLR2, 4 and 9 is also reported in the spleen and PBMC’s of VL patients. The findings may help to identify suitable TLR targets for TLR-based immune-therapy or TLR agonist-based *Leishmania* vaccines in the future [44].

### Nucleotide-Binding Oligomerization Domain (NOD) receptors

NOD-like receptors (NLRs) are a subset of pattern recognition receptors (PRRs) found in the cytosol that are essential for detecting invading pathogens and initiating the innate immune response. NLRs cooperate with TLRs and regulate inflammatory and apoptotic response and play key roles in the regulation of innate immune response [45]. Other NLRs, including NLRP1, NLRP3, and NLRC4, oligomerize to form multiprotein inflammasome complexes. Inflammasomes are central for cleavage and activation of pyrogenic cytokines, IL-1β and IL-18. NLRs and inflammasomes are linked to several autoimmune and inflammatory diseases as well as infections [46]. However, our knowledge of the roles of NLR in immunity against *Leishmania* infections is almost non-existent with the exception of the few studies on NLRP3. Despite all these advancements, our understanding on TLRs and cytoplasmic PRRs that recognize and respond to *Leishmania* is rather limited.

### Immune responses conferred by spleen, liver and skin

In visceral leishmaniasis, the clinical disease proceeds with fever, anaemia, emaciation and enlargement of the liver and spleen [47]. The spleen is infected in all cases of the disease and plays a central role in VL. The spleen becomes an evident site of interaction between the immune system and the *Leishmania* parasite, because all of the obligatory cellular and humoral participants in the immune response against the parasite are present in it in large quantities. In contrast to other organs like the liver, the spleen maintains the infection during the entire course of disease [48]. Splenic neutrophils also play an important role in early control of parasite growth, but not in the liver. Neutrophil depletion at the beginning of *L. donovani* infection leads to increase in parasite burden. In clinical practice splenectomised patients develop more severe and often fatal disease. In fact, IL-10 and other cytokines produced by granuloma cells may provide conditions for the survival of *Leishmania* [49, 50]. The increase in the plasma cell population in lymphoid organs is a common finding in leishmaniasis, and it may be present at relatively early stages of the disease. However, in VL, several factors like the well-characterized polyclonal B-cell activation [51, 52] and production of cytokines (e.g. IFN-γ, IL-10 & IL-6) [53, 54] and chemokines (e.g. CXCL12) may contribute to plasma cell differentiation and retention in the red pulp of the spleen [55].

The resolution of disease in the liver is associated with the development of granuloma which is one of the key features of hepatic resistance [56]. These granulomas in the liver are attributed to the development of a Th1-dominated granulomatous response, characterized by the high IFNγ production by CD4+ T cells, initiated by IL-12 secreted from the DCs. IL-12 has also been shown to facilitate activation of iNKT cells in conjunction with TLR9 signalling [57] and TLR9 dependent IL-12 production by DC [58]. However, as SIRP a–CD47 signalling inhibits DC maturation and IL-12 production [59]. The spleen is also reported to be the source of *Leishmania*-specific T lymphocytes that migrate to the liver, where parasite replication is highly active. In the liver, these pre-activated cells become effector T lymphocytes. In recent years, there has been plenty of evidence defining immune responses of specific organs/tissues during *Leishmania* infection. However, much of this information is derived from murine models [60] and the findings of murine studies may not necessarily be translated into human systems [61]. Hence, there is a dearth of knowledge regarding the complex mechanism that the parasite utilizes to evade the immune system in these organs.

Healthy skin contains a high number of patrolling and resident immune cells that potentially could drive a tissue resident immune response without the involvement of lymphoid tissues. This localized lesion provides a model for studying complex interactions of parasite species, host immune potential and genetic factors on which the outcome of infection depends. From an immunological point of view, the skin contains a network of immune cells designed to sense infection and control inflammation. The predominant immune cells in the human epidermis are Langerhans cells (LC), a highly specialized type of dendritic cells (DC) only present in the skin and skin-draining lymph nodes [62]. VL and DCL are associated with impaired T cell response against parasite antigens. In contrast, patients with CL and MCL have a strong type 1 immune response to a soluble *Leishmania* antigen (SLA). Besides, the T cell response of PBMCs from individuals with MCL is not appropriately modulated by IL-10 and TGF-β [63]. Cutaneous leishmaniasis usually leads to self-healing disease with life-long immunity against re-infection. Resolution is characterized by the induction of specific IFN-γ releasing CD4+ T cells [64]. Failure to cure is associated with elevated levels of IL-4 with low IFN-γ responses from *Leishmania*-specific CD4+ T cells [65]. IL-10 is one of the cytokines that downregulate inflammatory response, the reduced expression of its receptor would impair IL-10 ability to downregulate immune responses in MCL lesions, explaining the intense inflammatory infiltrates and tissue damage observed in this disease form. High levels of TNF-α are also detected in the sera from MCL patients during active disease, and the levels decrease after therapy [66]. Increased expression of IL-10 in *L. major* lesions was found to be associated with progressive disease [67]. The immune mechanism of post-kala-azar dermal leishmaniasis is different and obscure as immunobiology of the Sudanese and South Asian PKDL is not similar and is not well understood [68, 69]. The PKDL patients have distinct patterns of immunity in the skin and blood circulation. In the skin, immunity is regulated by IL-10 and FoxP3 [69, 70], because despite the enhanced levels of IFN-γ and TNF-α, their respective receptors are downregulated [71]. Additionally, in the skin, there is an increased presence of Th17 cells and IL-17 [70, 71]. By contrast, peripheral immunity is controlled mostly by CD8+ T cells that are the major sources of IL-10 and are anergic in nature [68]. However, the decreased frequency and secretion of CD26R promotes disease progression in Indian PKDL [72]. Although cellular infiltration is common in South Asian and Sudanese PKDL, the occurrence of granulomas is absent in the former, suggesting that granuloma formation in the latter accounted for its self-resolving nature [73]. Recently Mukherjee et al. studied and delineated the lesional immunopathology in terms of granuloma formation, Langerhans cells, tissue macrophages along with mRNA expression of IL-12, p40 and IL-10 [72]. Immunological studies conducted so far indicate that PKDL is not a localized disease, but involves complex systemic immunity and parasitic factors [68]. However, more studies would be needed to understand the role of immune cells, the mechanisms that regulate their antigen presentation and pathogen factors that influence antigen presentation and subsequent activation of the immune system.

### Quest for vaccine against leishmaniasis

**Fig. 1:**
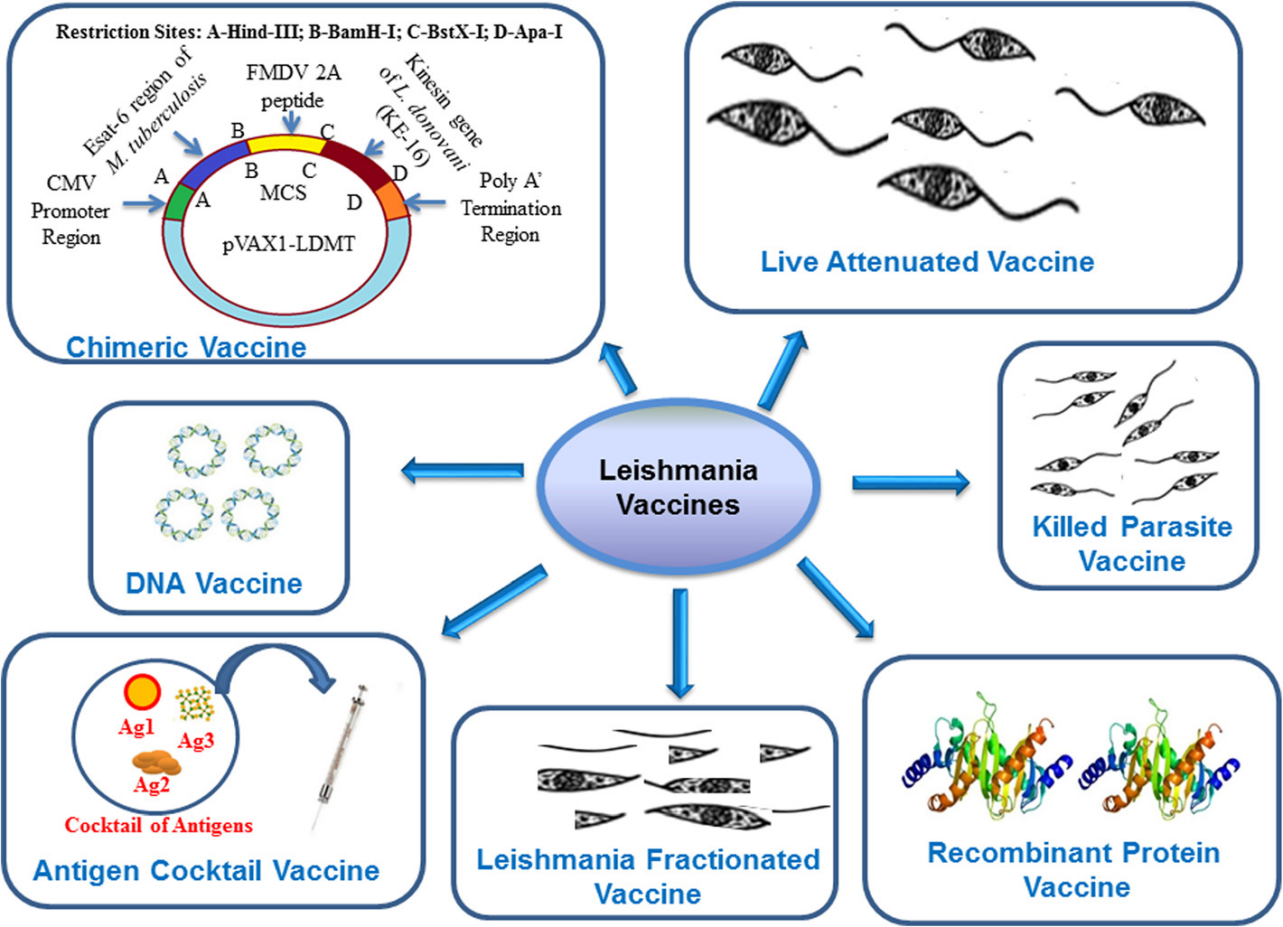
Vaccines against leishmaniasis

Although the geographical distribution of leishmaniasis is restricted to particular regions of the world, the public health concern caused by the infectious parasite is noteworthy. The disease is endemic in 88 countries, and as such, there is a considerable number of migratory population with around 350 million individuals being at risk, the impact is global. As such, current measures to address this concern include advanced surveillance, development of technologies for rapid assessment, production of drugs for improved treatment, vector control and identification of animal reservoir [73]. In addition to these important public health interventions, there has also been an international endeavour to develop a multispecies *Leishmania* vaccine [74]. At present there are no vaccines approved for general use; however, research and development is still underway. To date, there have been numerous attempts to develop a successful vaccine against leishmaniasis and there are several types of vaccine candidates but mostly prophylactic (Fig. 1). In general, the vaccines for the prophylaxis of leishmaniasis, which are in developmental stage, can be divided into three categories: (i) live attenuated *Leishmania* vaccines, including new genetically modified strains; (ii) killed parasite vaccines consisting of whole killed *Leishmania* or fractions of the parasite; and (iii) defined vaccines, i.e. recombinant proteins, DNA vaccines and combinations thereof (Table 1).

**Table 1:** Vaccines used for leishmaniasis

Antigen	Disease	Vaccine type	Animal model	Result/Outcome	Reference No. (year)
Centrin deleted live parasite *L. donovani*	VL	Live (Genetically modified)	Dogs	Protection	[76] (2015)
Ascorbic acid-deficient live mutants of *L. donovani*	VL	Live (Genetically modified)	Mouse	Protection	[77] (2015)
*Leishmania* parasites lacking essential genes like dyhydrofolate reductase, biopterin reductase and cystein proteases	CL (*L. max*)	Live (Genetically modified)	Mouse	Partial protection	[79] (2015)
	CL (*L. m.*)				
p27 gene knockout *L. donovani* parasites	VL (*L. d.*)	Live attenuated	Mouse	Protection	[82] (2013)
*L. major* (ALM) + BCG	VL & PKDL	Killed vaccine	Mouse	Partial protection	[5] (2006)
KBMA *L. infantum* and *L. chagasi*	VL	Killed vaccine	Mouse	Partial protection	[86] (2012)
Leishmune (FML)	VL (*L. d.*)	Fractioned vaccine	Dogs	Partial protection	[88] (2006)
KMP 11	CL (*L. m.*)	Recombinant vaccine	Hamster	Protection	[94] (2005)
Leish F1	VL (*L. d.*)	Recombinant protein	Clinical trial	Protection	[92, 93] (2011)
Leish 111f	VL (*L. d.*)	Recombinant vaccine	Mouse	Protection	[99] (2007)
HASPB1	VL (*L. d.*)	Recombinant protein	Mouse	Partial protection	[95] (2000)
Leish-f1+MPL-SE	VL (*L. I*)	Recombinant vaccine	Clinical trial	Protection	[93] (2011)
rSMT	VL (*L. d.*)	Recombinant vaccine	Mouse	Protection	[102] (2007)
A2	VL (*L. i.*)	Recombinant vaccine	Mouse	Partial protection	[101] (2009)
Leish-110f	VL (*L. i*)	Recombinant vaccine	Mouse	Protection	[98] (2009)
LD91+LD72+ LD51+LD31	VL (*L.d.*)	Antigen cocktail vaccine	Mouse	Partial protection	[109] (2009)
(P-8 PGLC)	*L. pifanoi*	Antigen cocktail vaccine	Dog	Partial protection	[110] (2007)
rCDV-LACK, rCDV-TSA, and rCDV-LmSTI1	CL (*L. m.*)	Antigen cocktail vaccine	Dog	Protection	[113] (2015)
Gp63	VL (*L.d.*)	DNA vaccine	Mouse	Partial protection	[119] (2011)
TRYP	VL (*L. i.*)	DNA vaccine	Dog	Protection	[121] (2009)
NH36	VL (*L. d.*)	DNA vaccine	Mouse	Partial protection	[123] (2009)
Proteophosphoglycans (PPGs)	VL (*L. d.*)	DNA vaccine	Hamster	Partial protection	[124] (2009)
ORFF	VL (*L. d.*)	DNA vaccine	Mouse	Partial protection	[127] (2009)
LPG 3	VL (*L. i.*)	DNA vaccine	Mouse	Partial protection	[128] (2014)
LEISHDNAVAX	VL (*L. i.*)	DNA vaccine	Mouse	Protection	[130] (2015)

*Abbreviations*: *VL* Visceral Leishmaniasis, *CL* Cutaneous Leishmaniasis, *PKDL* Post kala-azar dermal leishmaniasis, *L.d. L. donovani*, *L.i.*, *L. infantum*, *L.m. L.major*, *L.max*, *L. maxicana*

### Live attenuated vaccine

Live attenuated or first generation vaccine includes the inoculation of attenuated parasites. The development of a new vaccine must meet several strict criteria where safety, reproducibility and efficacy are of utmost importance [3, 5, 75, 76]. It is an appealing approach as attenuated parasites closely mimic natural infection that may lead to similar immune responses, without the danger associated with infection with live virulent parasites. Several procedures have been used to develop live attenuated *Leishmania* parasites, including long-term in vitro cultures, selection for temperature sensitivity, chemical mutagenesis and irradiation [75, 76]. This approach known as leishmanization, used only in one country, Uzbekistan, a mixture of live and dead *L. major* is licensed as a vaccine for high-risk populations [75]. The parasite is isolated from an active lesion to produce the vaccine each year to overcome the problem of loss of virulence. Although such live attenuated vaccines have shown substantial protection against challenge in animal models, undefined random genetic mutations and concerns arising from potential reversion to virulence makes such vaccines unsuitable for human vaccination. Indeed, the persistence of asymptomatic infection, especially in immune-compromised individuals, raises the risk of reversion to clinical disease. Moreover, attenuations due to undefined genome alterations can reduce effective protective immunity, either they fail to persist long enough to elicit an immune response or lack critical epitopes to evoke the protective response [76]. However, such mutants cannot be used as vaccine candidates, because they still carry wild-type alleles and could cause disease, especially in immunosuppressed individuals.

Another approach is the attenuation of the *Leishmania* parasites by addition of suicide cassettes that lead to the death of the parasite in response to external stimuli, an example of which is the introduction of drug-sensitive genes such as cytosine de-aminase gene, which is sensitive to 5-fluorocytosine [75]. In animal experiments it has been suggested that parasites carrying drug-sensitive cassettes could provide suitable candidates for leishmanization as an effective treatment of non-resolving lesions could be guaranteed. But no such formulation has reached even in pre-clinical stage as yet. In a clinical trial of live vaccine, in Iran, several live *Leishmania major* stabilates were produced from the seed lots for challenge studies. The stabilates were made from the late stationary phase of parasites in culture and were kept frozen in liquid nitrogen, until used. Two leishmanization trials were conducted in 42 inoculations in 28 male adult volunteers who were followed until complete healing of their lesions [5]. However, introduction of live attenuated *Leishmania* vaccine is not possible in an area with a risk of HIV infection.

Recently, live attenuated *L. donovani* centrin deleted parasites have shown to elicit protective immunity against leishmanial infection in mice and hamster models [76]. Some researchers also have used genetically engineered ascorbic acid-deficient live mutants of *L. donovani* which induced long-lasting protective immunity against visceral leishmaniasis [77]. Genetically modified *Leishmania* parasites lacking essential genes like dyhydrofolate reductase, biopterin reductase or cysteine proteases have been shown to stimulate protection against challenge with virulent parasite strains [78, 79]. However, for the live attenuated parasite vaccines, the primary barrier against widespread use remains the absence of clear biomarkers associated with protection and safety. Growth arrested live attenuated *Leishmania* parasite amastigotes have been used as a tool to develop vaccine candidates against VL [80]. It has also been reported that the combination of live attenuated parasite along with the cysteine proteinases and sand fly salivary antigen provide enhanced the protective efficacy [81]. Long-term protective immunity in BALB/c mice was also observed by using the live attenuated *L. donovani* p27 gene knockout parasites [82]. Attenuated vaccines offer a novel approach to immunization against leishmaniasis. However, there are fears that the parasite may revert back to a virulent form, particularly in HIV-positive persons who might get a fulminant form of vaccine induced leishmaniasis. The asymptomatic infections of visceral leishmaniasis is another challenge as standard diagnostics methods involving parasite detection in tissue aspirates is morally and technically inappropriate for them; serology-based tests cannot distinguish between uninfected, antibody-positive individuals and asymptomatic infected individuals. DNA-based diagnostic assays appear to most precise but the low predicted parasite burden in the asymptomatic carriers is again an issue. Therefore, developing tools that can predict progression to VL disease is an urgent need to established enrolment criteria for vaccine trials as well as outlining end-points to assess the vaccine efficacy. We and others have earlier shown that a significant number of exposed individuals may remain asymptomatic for several months and finally a good percentage of these asymptomatic persons may self-heal the infection. If these persons are vaccinated and if they develop the disease at a later date, it will be herculean task for national governments and insurance agencies to settle the issues of compensation and media cry. Also, targeted deletion of essential virulence genes can result in complete destruction of the parasite or mutants that may not elicit protective immunity [83]. Moreover, the problems associated with live vaccines are standardization and quality control. Such problems are of least concern with the use of killed parasites for vaccine candidates.

### Killed parasite vaccines

Since the late 1930s, pioneering work of Brazilian scientists had demonstrated that killed parasites have efficacy both as therapeutic and prophylactic vaccines against CL and VL. The early trials with killed *Leishmania* as a vaccine were conducted in Brazil in the 1940s. Killed *Leishmania* is an appealing vaccine candidate in terms of its stable biochemical composition and antigenicity, low cost and safety, but the whole-parasite vaccine candidates tested do not confer significant protection against human leishmaniasis [84]. Over the ensuing decades, numerous preparations of killed parasites were tested, either alone or in combination with a variety of different adjuvants. Although displaying well-tolerated safety profiles, to date, no first-generation vaccine using killed parasites has demonstrated sufficient efficacy as a prophylactic vaccine to be used in widespread control programs [85]. Most vaccine studies focused on CL, and there have been no clinical trials of first-generation vaccines produced from and for visceralizing *Leishmania* spp. Killed parasite vaccines using an alum-precipitated autoclaved *L. major* (ALM) given with a BCG adjuvant had shown promise as vaccines for VL and PKDL [5]. A whole-cell vaccine approach using *L. infantum* and *L. chagasi* promastigotes has been used, after treating them with the psoralen compound amotosalen and low doses of UV radiation. This treatment generated permanent, covalent DNA cross-links within parasites and resulted in *Leishmania* organisms termed killed but metabolically active (KBMA). The initial results were highly encouraging [86]. The killed *Leishmania* vaccine has been applied for its immunogenicity in human and mouse models. The modern insights into antileishmanial immunity offered possible explanations for the failure of the first generation vaccines in the field and have important implications for the vaccination strategies against leishmaniasis. It has been demonstrated that the inoculation of killed parasites into immune mice leads to a loss of infection induced immunity. Recently, Thakur et al. studied the protective efficacy of freeze-thawed promastigote antigen of *L. donovani* along with various adjuvants against visceral leishmaniasis infection in mice [87]. As the killed parasite vaccine is conceptually simple and easy to produce in leishmaniasis endemic countries, at low cost. However, standardization of vaccine derived from cultured parasites is difficult and it has hindered commercial development efforts. In countries with a rudimentary biotechnology industry and poor cold-chain distribution system of vaccines, autoclaving of the killed vaccine is the recommended method of sterilization and preservation. However, autoclaving lowers the immunogenicity of the parasite by destroying most of the proteins. Also, as the site of administration affects the efficacy of a vaccine, it is important to investigate the most effective method and route of administration. Vaccination with killed *Leishmania* parasite does not mimic natural infection and is less immunogenic. No clinical trial of killed vaccines has demonstrated a significant level of protection for use of prophylactic vaccines. However, concerns remain regarding the feasibility of developing killed, whole-parasite vaccines, including the variation in results obtained from different fields and clinical trial sites in the past and potential difficulties in producing such a product to good clinical manufacturing standards. Therefore, while offering a safer and more stable alternative, killed parasite vaccines warrant further investigation.

### *Leishmania* fractionated vaccines

Leishmune, the first vaccine tried for canine visceral leishmaniasis, consists of a purified *L. donovani* fraction, named fructose mannose ligand (FML) and a saponin adjuvant [88]. FML has been characterized as a major antigenic complex of *L. donovani* and the main antigen in this complex is NH36, an essential enzyme involved in the construction of the parasite’s DNA [89]. Leishmune is considered a promising tool for the prevention of canine visceral leishmaniasis and, furthermore, its potential as a transmission-blocking vaccine is promising for the control of zoonotic visceral leishmaniasis [90]. Partially or fully purified *Leishmania* fractions/subfractions have been widely used in experimental models owing to their excellent immunoprotective properties. Membrane antigens of *L. donovani* promastigotes (LAg) entrapped in cationic liposomes (both positively and negatively charged) could induce significant levels of protection in mice [91]. However, difficulties in large-scale production, standardization of in vitro culture conditions and purification procedures are some of the issues raised regarding these vaccines to be considered for clinical trials against leishmaniasis. Therefore, focus has also been on recombinant proteins, polyproteins, DNA vaccines and dendritic cell vaccine delivery systems, as discussed in the following sections.

### Recombinant vaccines

Recombinant protein vaccines are produced by genetically engineered cells to produce antigenic proteins [5]. Various proteins have been tested as possible vaccine candidates. The first candidate to reach phase I and II clinical trials was LEISH-F1 from the Infectious Disease Research Institute (IDRI, Seattle, WA) [92]. LEISH-F1 is comprised of three proteins that are conserved across various *Leishmania* spp. including *L. donovani* and *L infantum/L. chagasi* [93]. Other antigens included kinetoplastid membrane protein-11 (KMP-11)-encoding construct, protected golden hamsters from both pentavalent antimony-responsive (AG83) and antimony-resistant (GE1F8R) virulent *L. donovani* challenges. Vaccinated hamsters showed reversal of T cell anergy with functional IL-2 generation along with vigorous specific anti-KMP-11 CTL-like response. This was the first report of a vaccine conferring protection to both antimony-responsive and resistant *Leishmania* strains, reflecting several aspects of clinical visceral leishmaniasis [94].

Recombinant hydrophilic acylated surface protein B1 (HASPB1), a member of a family of proteins expressed only in metacyclic and amastigote stages, has shown efficacy in an experimental mouse model of VL [95]. The BALB/c mice were vaccinated with NH36 recombinant protein and saponin followed by challenge with *L. chagasi* amastigotes. The DTH response to leishmanial antigen was significantly higher (70%) in vaccinees over the controls [96]. The protective potential of cysteine proteinase type III (CPC) has also been evaluated in BALB/c mice by using a prime-boost strategy [97]. To date, only one multicomponent vaccine, Leish-111F, has been assessed in clinical trials. Leish-111F is a single polyprotein composed of three molecules fused in tandem: the *L. major* homologue of eukaryotic thiol-specific antioxidant (TSA), the *L. major* stress-inducible protein-1 (LmSTI1), and the *L. braziliensis* elongation and initiation factor (LeIF) [6]. An optimized version, known as Leish-111F, has recently demonstrated strong immunogenicity and some protective efficacy against *L. infantum* in mice [98]. The Leish-111F vaccine is moving forward into clinical trials as LeishF1 and has been put on trial in combination with the MPLSE adjuvant. This adjuvant consists of monophosphoryl lipid A, a potent TLR4 agonist, formulated with the antigen as a stable emulsion. A recent small-scale clinical trial in a *L. donovani* endemic area showed Leish-F1-MPL-SE was safe and well tolerated in people with and without prior VL exposure and induced strong antigen-specific T cell responses [92, 93]. Immune response and protection induced by Leish-111F formulated with monophosphoryl lipid A in a stable emulsion (Leish-111F_MPL-SE) and demonstrated that mice developed strong humoral and T-cell responses to the vaccine antigen. Leish-111F/MPL-SE is the first defined vaccine candidate to progress to human phase-I and phase-II clinical trials in healthy volunteers in South America, CL and ML patients in Brazil and Peru and patients cured of VL in India [93]. Analysis of the cellular immune responses of immunized, uninfected mice demonstrated that the vaccine induced a significant increase in CD4 T cells producing gamma interferon, IL-2, and tumour necrosis factor cytokines, indicating a Th1-type immune response [99]. Experimental infection of immunized mice and hamsters demonstrated that Leish-111F_MPL-SE induced significant protection against *L. infantum* infection, with reductions in parasite loads of 99.6%, a level of protection greater than that reported for other vaccine candidates in animal models of VL. Protective and immunostimulatory effect of the prime-boost pORT-LACK/MVA-LACK vaccination tested in a canine experimental model. Vaccination induced a reduction in clinical signs and, in parasite, burden in the liver, an induction of the *Leishmania*-specific T cell activation, as well as an increase of the expression of Th1 type cytokines in PBMC and target organs. The recombinant *L. major* H2B protein and its amino-and carboxyl-terminal regions were evaluated for their ability to induce cellular responses in LCL-cured subjects in order to assess their immunogenicity and their potential use as vaccine candidates against cutaneous and visceral leishmaniasis [100]. A recombinant *L. tarentolae,* expressing the *L. donovani*-specific A2 protein, was used as a vaccine against *L. infantum* infection in BALB/c mice and provided evidence that intravenous (*i.v*.) and, to a larger extent, intraperitoneal (*i.p*.) immunization with the recombinant *L. tarentolae*-A2 strain elicited favourable immune responses and significant levels of protective immunity against *L. infantum* infectious challenge [101]. The *L. infantum* sterol 24-c-methyltransferase (rSMT) has been reported as a protective vaccine against experimental VL [102]. Four new antigenic proteins S4, S6, L3 and L5, located in *Leishmania* ribosomes have been characterized as the prophylactic properties of these proteins were first studied in the experimental model of cutaneous leishmaniasis caused by *L. major* inoculation into BALB/c mice. The administration of two of them, LmL3 or LmL5 combined with CpG-oligo-deoxy-nucleotides (CpG-ODN), was able to protect BALB/c mice against *L. major* infection [103]. A cellular immune response to PpSP15, a protein from the sand fly *Phlebotomus papatasi*, was used as a vaccine for *L. major* infection in mice in combination with CpG as a prime-boost. Modality confers strong protection against *L. major* infection [104]. However, the experiments are currently in progress to investigate if the changes in the immune response after infection with the mutant parasite could provide any protection against the challenge with the wild type parasite. Saljoughian and colleagues used recombinant *L. tarentolae* expressing the *L. donovani* A2 antigen along with cysteine proteinases [CPA and CPB without its unusual C-terminal extension (CPB -CTE)] as a tri-fusion gene. It showed that immunization with both prime-boost A2-CPA-CPB(-CTE)-recombinant *L. tarentolae* protects BALB/c mice against *L. infantum* challenge [105]. Immunogenicity of Leish-Tec®, an A2-based vaccine for visceral leishmaniasis, has been studied in a heterogeneous canine population by evaluating induced antibody responses [106]. The immunization with the *L. infantum* recombinant cyclophilin protein-1 confers partial protection to subsequent parasite infection and generates specific memory T cells [107]. The role of immuno-stimulatory proteins of *Leishmania* has been used as a potent candidate for vaccine development against leishmaniasis [108].

### Antigen cocktail vaccines

Present progress to design vaccines agent against VL is also based on molecularly defined antigens, the second-generation vaccines. A large number of leishmanial antigens against experimental leishmaniasis have been attempted for vaccination; antigens LD91 (91-kDa), LD72 (72-kDa), LD51 (51-kDa) and LD31 (31-kDa) entrapped in cationic liposomes as the adjuvant can be potential components of future anti-leishmaniasis vaccines [109]. The P-8 proteoglycolipid complex (P-8 PGLC), an amastigote antigen of *L. pifanoi*, has been demonstrated to induce protection in the experimental model of canine visceral leishmaniasis [110]. The ability of 6 pre-clinical vaccine candidates have been evaluated to stimulate peripheral blood T cells of cured VL patients by measuring, the release of Th1 and Th2 cytokines. Such antigens include kinetoplastid membrane protein-11 (KMP11), sterol 24-c-methyltranferase (SMT), A2, cysteine proteinase B (CPB), K26/HASPB and nucleoside hydrolase (NH). Furthermore, these antigens produce a Th1- type immune response, suggesting that they may elicit good protection [111]. The identification as vaccine candidates of *Leishmania* antigens that elicit appropriate immune responses in the canine model is a key step in the rational approach to generate a vaccine for human visceral leishmaniasis [112]. Recently, a cocktail of rCDV-LACK, rCDV-TSA, and rCDV-LmSTI1, respectively, has been evaluated in dogs and found markedly protective [113].

### DNA vaccine

Most vaccine studies aim to limit parasite replication in the vertebrate host and this approach has been appreciated but also has some limitations. Over the past two decades, several investigators have searched for genes encoding leishmanial proteins that induce protection against CL and VL in experimental mode. DNA vaccines have leapfrogged from scientific curiosity to one of the most dynamic fields of research and may offer new alternatives for the control of infectious diseases. DNA vaccines induce a complete immune response against the encoded antigen [114]. DNA vaccines represent one of the most recent innovations in the field of immunization. DNA vaccines have been shown to induce a preferentially Th1 immune response, which is necessary for the elimination of intracellular parasites and are thus a promising strategy to control leishmaniasis [115]. Since the immune defence against *Leishmania* infection and cure from disease involves adaptive and innate cellular immune responses, it is perceivable that immunity depends on pathogen-specific CD4+ and CD8+ T cells with regulatory as well as effector functions of phagocytic effector cells [116, 117]. The *Leishmania* homologue for the receptors of activated C kinase (LACK), is the most extensively studied DNA vaccine against both cutaneous and visceral leishmaniasis, but it has shown inconsistent results. Heterologous prime-boost with gp63 antigen, with CpG-ODN as adjuvant, provided durable protection against *L. donovani* challenge in an experimental mouse model and was associated with robust cellular immune responses [118]. As gp63 is a major surface protein present in both amastigote and promastigote forms and shows a high homology between VL species [119], Intramuscular vaccination of foxhounds with a *Leishmania* multicomponent (10 antigens) DNA vaccine resulted in antigen-induced lymphoproliferative and IFN-γ (but not IL-4) responses in primed dogs. The response was parasite-specific type 1 and it was able to restrict parasite growth [120]. A prime/boost DNA/modified vaccinia virus Ankara vaccine expressing recombinant *Leishmania* DNA encoding TRYP is safe and immunogenic in outbred dogs; it showed antigen-specific type-1 responses and in vivo memory phase cellular immune responses, consistent with superior potential for protective vaccine [121]. Immunotherapy against VL with the nucleoside hydrolase-DNA vaccine of *L. donovani* was carried out and the vaccine was highly effective as a new tool for the therapy and control of VL [122]. The NH36 gene of *L. donovani*, as a DNA vaccine, followed by the FMLSAP vaccine, induce significant cross-protective response against tegumentary leishmaniasis induced by *L. amazonensis*, indicating its potential use as a bivalent vaccine against visceral and tegumentary leishmaniasis. The polytope approach of genetic immunization is a promising strategy for the prevention of infectious disease as it is capable of generating effective cell mediated immunity by delivering the T cell epitopes assembled in the series [123]. *Leishmania* produces several types of mucin-like glycoproteins called proteophosphoglycans (PPGs), the DNA-encoding N-terminal domain of the *ppg gene* was evaluated as a vaccine in golden hamsters (*Mesocricetus auratus*) against the *L. donovani* challenge. The prophylactic efficacy to the tune of 80% was observed in vaccinated hamsters and all of them could survive beyond 6 months, after challenge. The efficacy was supported by a surge in inducible NO synthase, IFN-γ gamma, TNF-α and IL-12 mRNA levels, along with extreme down-regulation of TGF-β, IL-4, and IL-10. A rise in the level of *Leishmania*-specific IgG2 was also observed, which was indicative of enhanced cellular immune response [124]. Non-coding pDNA bearing immune stimulatory sequences co-entrapped with leishmanial antigens in cationic liposomes elicits almost complete protection against experimental visceral leishmaniasis in BALB/c mice [125]. An additional advantage of DNA vaccines is their potential as therapeutic vaccines, aimed at reinforcing or redirecting the immune response of an infected host to control disease progression [126]. The ORFF protein is present in both promastigote and amastigote forms of the parasite, but is preferentially expressed in *L. donovani* amastigotes [127]. Ubiquitin conjugation of ORFF DNA vaccine leads to improved cell-mediated immune response and induces protection against both antimony-sensitive and resistant strains of *L. donovani* [127]. Lipophosphoglycan which is essential for the synthesis of glycoconjugates as parasite virulence factors have also been used for the evaluation of the immunogenicity of *L. infantum* LPG3 gene as a DNA vaccine against murine visceral leishmaniasis [128]. The *L. amazonensis* experimental infection has been analysed by using DNA vaccine encoding iron superoxide dismutase [129]. LEISHDNAVAX, a multi-antigen T-cell epitope-enriched DNA vaccine, has been proved immunogenic and showed prophylactic efficacy in preclinical studies. Riede et al. described the safety testing of LEISHDNAVAX in naïve mice and rats, complemented by the demonstration of tolerability in *Leishmania*-infected mice [130]. The study indicated a favourable safety profile of LEISHDNAVAX in both naïve and infected animals and thus, supports the initiation of clinical trials for both preventive and therapeutic applications of the vaccine. Tabatabaie et al. investigated the protective efficacy of TSA-based DNA vaccine against *L. major* infection and it induced Th1 platform immune response and aluminium phosphate could improve the efficacy with induction of humoral and cellular immune responses against *L. major* infection [131]. In susceptible BALB/c mice, DNA vaccination with the preparation, encoding Lipophosphoglycan 3 of *L. infantum* showed elicit robust parasite-specific protective Th1 responses [128].

DNA vaccines present a multitude of advantages over other vaccine strategies and several features have made them an appealing alternative [4]. DNA vaccines may provide better protection against *Leishmania* than killed or live-attenuated vaccines as they can induce the expression of *Leishmania* antigens, which are unaltered in their protein structure and antigenicity [4]. However, delivery of DNA vaccines is an important concern. Vaccination with plasmid DNA encoding protective *Leishmania* antigens gives a promising approach to vaccination against leishmaniasis in that it has intrinsic adjuvant properties, induces both humoral and cell-mediated immune responses and results in long lasting immunity. However, the DNA vaccines for human trial are still awaited eagerly as none of those shown promising effects as per the clinical trial.

### Chimeric DNA vaccines

In the aspect of developing an anti-leishmanial vaccine, our laboratory has been working on immunological potential of the kinesin motor domain region of the *L. donovani* as a potential vaccine candidate against VL [132]. The kinesin motor domain of *L. donovani* is also a member of the kinesin protein superfamily. In *L. donovani,* it has been found to play an important role in cell division and intracellular transport of various cargoes including vesicles, organelles, large protein complexes and cytoskeletal filaments and is highly conserved in nature, at the nucleotide sequence level. Our laboratory has characterized the kinesin gene from two Indian isolates of *L. donovani* (GenBank acc. no. AY615886 and AY615887). The sequencing results revealed that the kinesin gene is ~ 3,000 bp long, comprising of one long open reading frame (kinesin motor domain), followed by immune dominant repeat region with 4–6 tandem repeats of 117 bp [133]. It has been reported that about 20% VL patients will also have tuberculosis in TB high burden countries like India. The *esat-6* gene of *M. tuberculosis* has earlier been shown to have diagnostic [Patent # WO 2010/010577 A] as well as immunogenic potential against tuberculosis and likewise kinesin motor domain has vaccine potential against visceral leishmaniasis. Both organisms are intracellular pathogens leading to chronic infection and have several similarities. Therefore, we developed a novel chimeric DNA vaccine candidate comprising the *esat-6* gene of *M. tuberculosis* and kinesin motor domain gene of *L. donovani* [134]. Both genes were cloned together on two sides of self-cleaving peptide in a DNA vaccine vector pVAX-1 wherein the chimeric construct is operatively linked to a transcriptional promoter thus capable of self-replication and expression within the mammalian cell. Further, immunogenicity of chimera DNA vaccine was also studied and the immune response compared to individual as well as chimeric clones in a mice model. The chimeric DNA construct generated cellular immune response with significant increase in IFN-γ and IL-2 cytokine levels, which was an indication of Th1 immune response (*P* < 0.05). Whereas the level of the IL-4 and IL-6 markers of Th2 response, were found comparatively lower. Our result of cellular immune response suggested that, our novel chimeric DNA vaccine could be as a potential immunoprotective candidate and have important implications in future vaccine design. This may offer an attractive alternative strategy against leishmaniasis and tuberculosis co-infection [134].

### Recent vaccine field trials

The transition from laboratory testing for evaluation in the field condition is a significant phase in the development of vaccine against any disease. There are a range of issues coupled with this transition, including safety and benefit to the patient or community. These limitations raise complex human ethical issues in the quest for successful vaccine development. Furthermore, a careful understanding of defensive immune prophylaxis and immunological memory maintenance is equally significant in vaccine trials.

Development of a successful vaccine to prevent kala-azar has been an objective for nearly a century, but as of now, no such vaccine has reached to the level of clinical use. The only vaccine, which has reached to clinical trials is Leish-111F [5, 92]. There is some evidence that the Leish-111F vaccine can also induce partial protection against visceral leishmaniasis in animal models. However, Leish-111F failed to protect dogs against infection and did not prevent disease development in a recent phase III trial in dogs. Human Phase I and II clinical trials (safety and immunogenicity) of Leish-111F has been completed over the past few years in Brazil, Peru and Columbia, and Phase I trial has been conducted in India [135].

### *Leishmania* vaccine: a challenge may become a reality

For the controlling of any infectious disease, it is the vaccination, which can be used as a cost-effective mean. Various vaccines have successfully been used for controlling various infections, and even to complete eradication of diseases like smallpox and polio. Like other infectious diseases, leishmaniasis also ought to be controllable by vaccination in view of the body of evidence from studies in humans and animal models. Yet, no vaccine is currently in the market despite much effort. Therefore, the question arises what are the reasons or limitations in developing a successful anti-leishmanial vaccine? In our opinion, some of the key issues are:

1. Marginal or no profitable recovery from the market:
  Vaccines against infections of the 3rd World such as schistosomiasis, malaria, leishmaniasis and several viral and bacterial diseases are unattractive to the industry in view of financial profits, since the market is not sufficiently lucrative to recover the cost of the development (300 to 800 million US dollars per vaccine development program) [136]. WHO and several charitable foundations, such as the Bill and Melinda Gates Foundation, contributing greatly to the development of anti-parasitic vaccines and leishmaniasis is on their list. Public-private partnerships have also been suggested and the idea seems to have been taken on board by Big Pharma companies.
2. Failure in conferring the immunity:
  Soon after the *Leishmania* infection individuals are considered to have acquired long-lasting immunity to infection with the similar parasite, making vaccination a practical measure. However, despite of many promising vaccine candidates, developing a vaccine against human leishmaniasis is still difficult. The killed vaccine candidates have concerns of lot viability and good protective immunity, the live attenuated vaccines have the danger of vaccine induced leishmaniasis in immunosuppressed individuals like AIDS patients. The recombinant and DNA vaccines are in their initial phases and more data are yet to come. Side effects are another issue with these genetically modified vaccine candidates.
3. Suitability of adjuvant:
  Development of an effective vaccine requires precise information about the adjuvant to be used and the specific formulation which makes it stable, safe and immunogenic. The suitability of the adjuvant depends on factors such as the nature of antigen (associated/co-administered), the route of administration, the immunization schedule, and the type of required immune response. However, these parameters vary on a case to case basis in order to develop an effective vaccine.
4. Differences in virulence dynamics of the *Leishmania* species:
  The clinical manifestation of *Leishmania* infection is diverse, ranging from the cutaneous to visceral form. Within these categories, *Leishmania* infection is able to produce a large variety of atypical and rare variants. The diversity is due to variation in the causative species of *Leishmania* of New World and Old World. For example, despite causing cutaneous disease, the Old and New World parasites, *L. major* and *L. mexicana/L. amazonensis*, respectively, are markedly different. Phylogenetic analysis has revealed that *L. major* is as distantly placed from *L. mexicana/L. amazonensis* as it is from *L. donovani*, which causes an entirely different disease. There are differences in virulence factors among these species as well as in the immune responses that they induce. These major problems are currently hampering the anti-leishmanial vaccine development and implementation. The ethnicity of the host is another important confounding factor.

## Conclusion

Leishmaniasis is a group of different manifestations and is the foremost cause of morbidity and mortality, throughout the world. The preventive vaccines are recognized as the best and most cost-effective protection measures against infectious pathogens, and theoretically *Leishmania* should not be the exception. Effective vaccine will be crucial to meet the target to eliminate leishmaniasis from the Indian subcontinent by 2020. In spite of the availability of various therapeutic agents with greater success and tremendous achievement in controlling the disease, there is no effective vaccine available for visceral leishmaniasis, the most severe form of the infection. *Leishmania* vaccine development has proven to be a difficult and challenging task, which is mostly hampered by inadequate knowledge of parasite pathogenesis and the complexity of immune responses needed for protection. The parasites activate the innate and adaptive arms of the immune system, and it is clear that a coordinated network of responses is required for effective immune-mediated parasite clearance. Success of vaccine development depends upon understanding the immunobiology of pathogen/host interactions, selection of appropriate vaccine candidates and choosing the right adjuvant or delivery vehicle. Currently, there seem to be many problems, even though we are getting closer to develop a safe and effective leishmaniasis vaccine(s). It is becoming possible with the help of newer technological advances and developments in the vaccinology field, which have evolved from whole irradiated live parasites, to the use of defined antigens such as LEISH-F, including the DNA vaccines. Therefore, our efforts must continue in the quest of safe and practical vaccine to eliminate this parasitic disease from the world.
